# Foresight and Preparedness Studies on COVID-19 in the World: A Systematic Literature Review: Mariana Peyroteo dos Santos

**DOI:** 10.1093/eurpub/ckac129.064

**Published:** 2022-10-25

**Authors:** M Peyroteo, J Vukovic, L Lapão

**Affiliations:** 1 Comprehensive Health Research Center, NOVA University of Lisbon, Lisbon, Portugal; 2 Croatian Institute of Public Health, Zagreb, Croatia

## Abstract

A foresight study refers to a broad range of methodologies to describe possible futures. Also, foresight can be described as ‘a university human capacity that allows people to think ahead and consider, model, create and respond to future eventualities’ (Foresight International, 2006). Foresight studies in public health are research tools to support the understanding of possible future developments, which is essential for policy makers to anticipate and possibly influence trends. Therefore, we aimed at providing an overview of COVID-19's foresight activities across Europe and beyond. A systematic literature review was conducted following Preferred Reporting Items for Systematic Reviews and Meta-Analysis Methodology. The databases searched were Scopus and Web of Science Core Collection and the literature screening was conducted through March 2022. The exclusion and inclusion criteria were previously defined by the team and all the results had to be journal papers, published between 2019-2021 (except for “Foresight” query that was between 2015-2021) and written in English or Portuguese. The documents collected were only about Population Health and Non-Pharmaceutical Approaches. Among the topics under study (foresight, scenarios, modelling and preparation) it was possible to collect 9 550 articles. After removing duplicates and filtering out those that did not meet the inclusion criteria, 2 434 articles were selected for analysis. The literature review revealed that a large number of studies on the pandemic of COVID-19 have already been conducted using predictive methodologies, but that the focus of attention is on analysing how countries are prepared for the pandemic and what direction it will stat using mathematical modelling methods (e.g. in the short term). This study highlights the importance of predictive studies and that their use needs to be well-founded and cohesive if the conclusions drawn are to have impact and value in the future.

